# Effect of High-dose Biotin on Thyroid Function Tests: Case Report and Literature Review

**DOI:** 10.7759/cureus.2845

**Published:** 2018-06-20

**Authors:** Arash Ardabilygazir, Sonia Afshariyamchlou, Danial Mir, Issac Sachmechi

**Affiliations:** 1 Medicine, Icahn School of Medicine at Mount Sinai Queen Hospital Center, New York, USA; 2 Internal Medicine, Icahn School of Medicine at Mount Sinai Queen Hospital Center, New York, USA

**Keywords:** high dose biotin, thyroid function tests, multiple sclerosis, graves disease, hyperthyroidism, biotin-streptavidin immunoassay

## Abstract

Biotin is a readily available supplement that is part of the B-complex vitamins. It is an essential co-factor for five carboxylases involved in fatty acid synthesis and energy production. The recommended daily intake (RDI) of biotin ranges from 30 to 70 mcg per day. At high doses (10,000 times RDI), biotin improves clinical outcomes and quality of life in patients with progressive multiple sclerosis (MS). It has been reported to cause interference in immunoassays resulting in abnormal thyroid function tests. Hereby we are describing the case of a patient having MS who was on high-dose biotin, seen in the clinic for a follow-up visit with thyroid function tests suggestive of Graves’ disease with no signs and symptoms of hyperthyroidism and completely normal physical examination. In the case we have described, the laboratory measurements suggestive of thyrotoxicosis were attributed to interference of the patient’s high-dose biotin treatment with the biotin-streptavidin chemistry of the immunoassays. We observed normalization of the thyroid stimulating hormone (TSH) and free T4 measurements when the patient withheld biotin for a week. As our case illustrates, early consideration of biotin interference minimizes unnecessary repeat laboratory studies. As trials in MS are progressing, we expect to see more patients on high-dose biotin treatment with spurious laboratory measurements. Therefore, we advise careful history taking and close communication with the laboratory when the clinical picture does not match with the laboratory results.

## Introduction

Biotin is a readily available supplement that is part of the B-complex vitamins. It is sold over-the-counter under a variety of names, including vitamin B7, vitamin H and coenzyme R, and sometimes may be an unnamed supplement advertised as an aid to improve hair and nail health.

The discovery of biotin occurred in response to a research conducted in 1927 that investigated the cause of what was called “egg white injury,” a vitamin-deficiency disease observed chiefly in animals and is induced by feeding on an excess of raw egg whites [1].

Biotin is an essential co-factor for five carboxylases involved in fatty acid synthesis and energy production. The recommended daily intake (RDI) of biotin ranges from 30 to 70 mcg per day. At high doses (10,000 times RDI), biotin improves clinical outcomes and quality of life in patients with progressive multiple sclerosis (MS). Biotin is a common component of multivitamin preparations and at high doses it has been reported to cause interference in immunoassays resulting in abnormal thyroid function tests [2].

High biotin doses are also prescribed for several rare inherited metabolic diseases (biotinidase deficiency: 5-10 mg/day; holocarboxylase synthetase deficiency: 30-40 mg/day; biotin-thiamine-responsive basal ganglia disease: 100-300 mg/day). Moreover, supraphysiological doses of biotin (up to 30 mg/day) are now widely used for self-medication aimed at reducing hair loss or improving nail or skin condition [3].

Hereby we are describing the case of a patient with MS who developed abnormal thyroid function tests on high-dose biotin.  

## Case presentation

A 49-year-old woman had a past medical history of MS; she was on post iodine-131 therapy for Graves' disease and was euthyroid after that for three years. In a follow-up visit, her thyroid function tests showed markedly elevated free T4 to 3.2 ng/dl and suppressed thyrotropin (thyroid stimulating hormone, TSH) to 0.08. She was treated with Copaxone (Teva Pharmaceuticals, PA, USA) 40 mg injection three times weekly. A month before this thyroid function test was done, the patient started to have 200 mg of biotin orally daily.

On physical examination, the patient’s thyroid gland was found to be normal and had no signs of Graves' disease. Thyroid function tests were repeated. Total triiodothyronine (T3) and free thyroxine (FT4) were both markedly elevated, while TSH was suppressed and the TSH binding-inhibiting antibody test was positive (Table 1). This combination of test results was suggestive of Graves’ disease. However, the laboratory data were in striking contrast with the paucity of signs and symptoms observed in the patient.

The patient was asked to stop the biotin treatment temporarily; and one week later, repeated thyroid function tests showed completely normal results (Table 1). No other change was made to her medication list, and she continued to feel well. Biotin treatment was resumed thereafter.

**Table 1:** Test results.

	11/3/2015 off biotin	5/19/2016 on biotin	6/1/2016 on biotin	6/7/2016 off biotin	Reference range
TSH	2.20	0.08	0.02	2.00	0.27-4.20 uIU/ml
Free T4	1.4	3.2	7.8	1.3	0.9-1.8 ng/dl
Free T3	2.80	3.92	7.38	3.05	1.80-4.60 pg/ml

## Discussion

**Figure 1:**
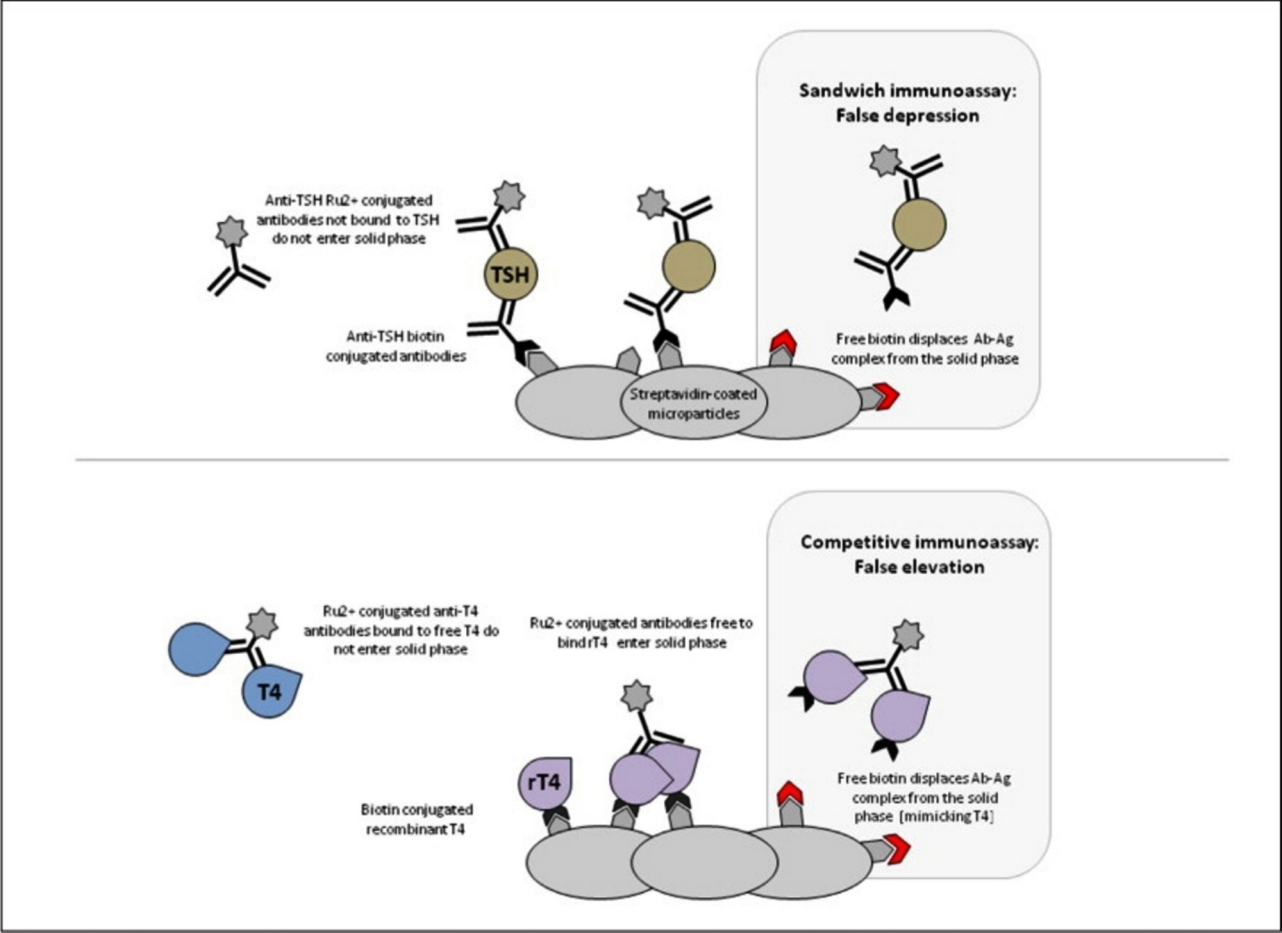
Roche system. Mechanism of biotin interference in Roche immunoassays. The Roche sandwich immunoassay employs a biotinylated antibody to the analyte (thyroid-stimulating hormone, TSH in this example); therefore, excess free biotin displaces antigen-antibody complexes (Ab-Ag), which leads to less ruthenium (Ru2+)-conjugated antibody binding to the solid phase and false depression of analyte measurement. The Roche competitive immunoassay utilizes a complex of biotinylated analyte (thyroxine [T4] in this example) and microparticles with recombinant T4 (rT4) that displace the sample analyte being measured from an antibody-conjugate with Ru2+. Excess free biotin also displaces Ab-Ag complex from the solid phase, producing false elevations in sample analyte measurements.

​​​​​This patient showed markedly abnormal thyroid function tests in the absence of the symptoms of thyroid dysfunction. All the abnormal tests were obtained with immunoassays employing the streptavidin-biotin immobilizing system. The complete revival of normal thyroid function tests just one week after the interruption of a megadose of biotin treatment suggests the latter as the cause of the biochemical abnormalities observed. Streptavidin, a protein produced by *Streptomyces avidinii*, also called avidin, binds biotin having a dissociation constant, which is thousands times higher than what most antibodies show for their specific haptens [4]. The TSH assay used for this patient employs a typical “sandwich” design. The serum sample is incubated with a mixture of a biotinylated monoclonal TSH antibody and a ruthenium-labeled monoclonal TSH antibody. The immune complexes formed between the two antibodies and the TSH in the serum are then captured by streptavidin-coated magnetic microparticles. The chemiluminescence produced upon application of a voltage will in this case be directly proportional to the TSH level in the serum sample. Excess biotin in the serum will result in reduced binding of the immune complexes with the solid phase, and hence a falsely low TSH level. This interference from biotin may be particularly intense in the Roche system [5] (Figure 1) in which the solid phase is generated only after the patient’s serum is added to the mixture. Other assays, in which the interaction between biotinylated antigens or antibodies and streptavidin is established before the incubation with the sample, may be more resistant to this interference.

The interference of pharmacologic biotin levels in immunoassays of hormones has been previously described in a few case reports (Table 2). Minkovsky et al. described a woman with a history of MS taking high-dose biotin with the same interference [5]. Kwok et al. described falsely depressed TSH in a girl taking high-dose biotin supplementation [6]. Wijeratne et al. demonstrated a similar effect after ingestion of 30 mg of biotin in a single subject [7]. Clerico and Plebani reported that healthy volunteers taking 15-30 mg/daily dose of biotin can actually show concentrations > 30 ng/mL, and so should present some significant interference in immunoassays, based on biotin-streptavidin technology [8]. In an Australian study from 2012, one of the authors ingested 30 mg of biotin, and blood samples were collected before intake and after 1, 2, 4, 8 and 25 hours. An increase in the measured concentrations of both free T3 and free T4 was present. Both concentrations peaked around two hours after ingestion, and the increase lasted for at least 24 hours [9].

**Table 2:** Summary of the reviewed cases.

First author (Ref.)	Year	Case	Biotin dose	Clinical consequences
Minkovsky	2016	A 74-year-old female	100 mg TDS	FT4 ↑ TSH ↓
Kwok	2012	A 3-year-old female	10 mg QID	FT4 ↑ FT3 ↑ TSH ↓
Wijeratne	2012	A 42-year-old male	30 mg single dose	FT4 ↑ peak interference at 2h, duration and magnitude varied according to analyte
Clerico	2013	A 50-year-old female; A 62-year-old male	15 mg OD; 30 mg OD	FT4 ↑↑ significant interference
Wijeratne	2012	A 1-week-old baby	10 mg TDS	FT4 ↑ TSH ↓

This case illustrates that prompt recognition of biotin interference requires a high index of suspicion because the interference mimics the laboratory changes in thyrotoxicosis. This patient required additional evaluation, including repeat blood tests and endocrine specialty visits. This additional evaluation added cost and inconvenience to both the patient and the overall medical system. Despite warnings from the laboratory assay manufacturer, most clinicians may not be aware of their laboratory’s particular assay or this specific potential interference by biotin. As not all laboratory assays use biotin-containing components, it is important to keep open communication between the laboratory and the clinical providers to ensure that clinicians are aware of this potential inference. Another impediment in recognizing the interference is that biotin may not be listed in the medical record as a medication. Specific ascertainment of dosage and preparation is imperative to correctly identify the source of interference.

## Conclusions

In our case, the thyroid function test result was suggestive of hyperthyroidism even though the patient did not have any signs or symptoms of it. This was attributed to the biotin-streptavidin immunoassays. We observed the normalization of TSH and free T4 when the patient withheld biotin for a week. As our case illustrates, early consideration of biotin interference minimizes unnecessary repeat laboratory studies. Also further investigations are needed to precisely identify the impact of biotin intake on tests using biotinylated antibodies.
